# Infrared Irradiation: Toward Green Chemistry, a Review

**DOI:** 10.3390/ijms17040453

**Published:** 2016-03-26

**Authors:** René Escobedo, René Miranda, Joel Martínez

**Affiliations:** 1 Department of Chemistry, Faculty of Superior Studies Cuautitlan, Campus 1, Autonomous National University of Mexico, Cuautitlan Izcalli, State of Mexico 54740, Mexico; renegerardo.escobedo@gmail.com (R.E.); mirruv@yahoo.com.mx (R.M.); 2 Chemistry Science Faculty, Sciences and Engineering Postgrade, Autonomous University of San Luis, San Luis Potosi, State of San Luis Potosí 78210, Mexico

**Keywords:** catalytic clay, clean energy, green chemistry, infrared irradiation, natural products extraction, reaction activation

## Abstract

This review provides a comprehensive overview of where infrared irradiation has been employed, mainly as regards activating green mode for natural products extractions, as well as to favor a reaction, highlighting its actual importance. It is also underlined that infrared irradiation heating has been around for a long time; however, only in the last eighteen years have many of its advantages been applied to satisfy a wide range of chemical processes, natural products extractions, and for the promotion of many kinds of reactions. In addition, it is brought to light that near infrared irradiation is more efficient than middle and far infrared irradiations, being easily controllable and with the quality of a fast responding heat source. Thus, the main objective of this review is to offer infrared irradiation as an alternative clean energy source to activate reactions, in addition to favor the selective extraction of natural products, all of which is within the Green Chemistry protocol. Some recent results from our laboratory are also included.

## 1. Introduction

Green Chemistry is an actual field that strives to work at the molecular level to achieve sustainability; since the early 1990s, this paradigm has acquired its up-to-date position as a scientific discipline, as well as a practical mode for the prevention of pollution.

Green Chemistry has a Protocol of a cohesive set of Twelve Principles [1]. In this regard, researchers at the vanguard of innovation must have the knowledge to design chemicals and chemicals manufacturing processes with little or no risk to human health or to the environment. However, no activity can be completely risk free, waste free or lower harmful emissions. Consequently the “Twelve Principles of Green Chemistry” should be viewed and applied as a scientific reflection [2].

Most chemical processes (75%) use thermal sources of energy that originate from fossil fuels, and the rest come from biomass and non-carbon sources [3]*.* It is worth noting that Principle 6 (energy requirement should be more recognized for their environmental and economic impacts, and they must be minimized) is one of the most ignored of the 12 principles of Green Chemistry. Thus, in order to minimize energy requirements, with a view to green chemistry, attempts are, and must continue being, made to make energy input in chemical systems as efficient as possible. Approaches have been taken and new possibilities are investigated; in this regard, some of the so-called non-classical energy forms include: sonication (US), mechanical, also known as tribochemistry or mechanical milling (MM), microwave (MW) and, more recently, the infrared irradiation (IR); all them employed to minimize reaction time, improve the product yield and avoid undesired byproducts. It is also worth mentioning that other classical methods exist: thermal-typical mantle heating (MH), photochemical and electrochemical [4].

Taking into account that the goal of this review is to bring to light the importance of infrared irradiation in the chemistry field and more particularly the Green Chemistry Protocol, it is worth noting that the infrared region in the electromagnetic spectrum is divided into three zones: shortwave infrared, also known as “near” or “high intensity” infrared with band spans from 0.76 to 2 µm (NIR); medium wave infrared also known as “middle” or “medium intensity” infrared with band spans from 2 to 4 µm (MIR); and long wave infrared also knows as “far” or “low intensity” infrared with band spans from 4 to 1000 µm (FIR).

Moreover, infrared energy is dispersed from an infrared emitter (lamp) and consequently exposes product surfaces, which easily absorb it and are heated. Therefore, heating effectiveness is related to the line of sight between the source and the product; in other words, infrared irradiation is a direct form of heating. This in addition to the promotion of vibrational modes in a molecule are the main reasons for the inherent high energy efficiency of infrared systems to activate a reaction or favor an extraction.

Emitters are specifically designed for different energy output characteristics [5]. For long wavelength (FIR), a resistance element is sandwiched in hardened glass or vitrified ceramic. It is in general considered a low temperature heat source since the resistance element has high mass, which can increase the temperature up to 1000 °F (540 °C), with peak wavelength 3–5 µm and response time 5 min. Related to medium wavelength (MIR), a chromium alloy filament is suspended in a quartz or metal sheath. The filament can operate in open air, increasing the temperature up to 1800 °F (980 °C), with peak wavelength 2.3 µm and response time 30 s. Finally, for short wavelength (NIR), a tungsten filament is sealed in a quartz envelope with a halogen gas. It is important to note that the thin filament, due to its little mass, is very reactive to the voltage applied. Consequently, NIR heat output changes immediately with the corresponding changes of the applied voltage, increasing the temperature up to 4000 °F (2200 °C), with peak wavelength 1.2 µm and response time <1 s. Thus, it is noteworthy that NIR offers many advantages over long and medium infrared wavelengths (*vide supra*).

In addition, the interaction of NIR with matter can be considered from classical mechanical model for a diatomic molecule. Thus, in an anharmonic model, the molecule is considered as two balls connected by means of a coil, that take into account several assumed behaviors: the repulsion among the electrical-neighboring of the approximated atomic nucleuses and the bond strength when the atoms move away from each other. Consequently, it can be considered that this tension, between the atomic cores, could cause the corresponding bond separation. The anharmonicity can also be present in the electrical properties of a molecule; it affects its dipole moment, which, in anharmonic model, does not have a linear dependence with the interatomic distance.

Now, it is not surprising that the NIR technology has found a vast field of application [6], some of the sectors where NIR technology has succeeded for qualitative and quantitative purposes are: Agricultural/Food (agricultural products, industrial food products, and precision agriculture/soil); Polymer (polymer processing and polymer quality characteristics), Petroleum and Fuel Industry (fuel quality control, fuel production process, and petroleum characterization), Environmental, Textiles, Biomedical/Clinical, Pharmacy and Cosmetics, and NIR-image.

Taking into account all previous commentaries, it is worth mentioning several innovative modes favoring the activation of a chemical reaction, other than conventional mantle-heating, have been developed. The use of the mechanical ultrasound waves, mechanochemistry, microwave and infrared electromagnetic irradiations have earned increasing attention in the Green Chemistry protocol, according Principle 6. Thus, MW, US and MM, under controlled conditions, are invaluable technologies with enormous applications in academic and industrial research. However, the successful use of these methodologies is limited by requiring access to specific and expensive equipment [7]. Consequently the goal of this Review is to offer an overview of works where IR has been successively employed, and sometimes in comparison with other technics; for example, microwaves are quite useful and appear quicker than IR; however, in general, the difference is not significantly important and occasionally this is not the case. In other words researchers developing IR reactions are able to obtain very good work conditions for their reactions.

Thus, the main goal of this review is to offer a comprehensive overview of where “green” infrared irradiation has been employed as the activating mode both for natural products extractions, as well as to favor a reaction, highlighting its actual importance in the chemistry field and more particularly in the Green Chemistry Protocol; therefore, the results of a wide literature search are summarized in the following sections.

## 2. Related Aldol Condensation Reactions

The well-known condensation of Knoevenagel, a base or acid catalyzed aldol-type reaction, is generally raised between aromatic aldehydes as substrate and a methylene active reagent [8]; this interesting protocol has been promoted in many cases by the use of middle infrared irradiation.

**Scheme 1:**
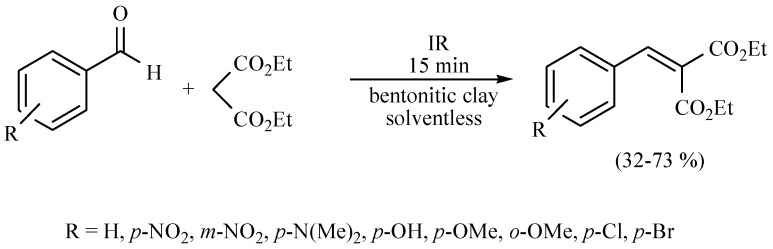
Synthesis of benzylidenemalonates.

**Scheme 2:**
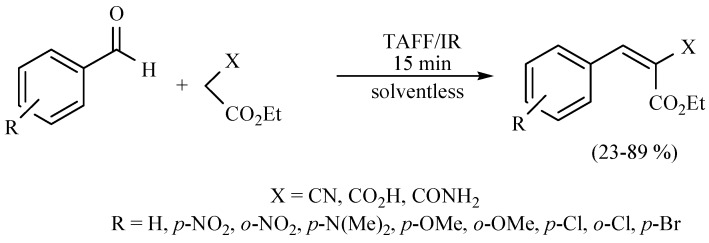
Synthesis of benzylidenemalononitriles, benzylidenecyanoacetamides, and benzylidenecyanoacetic acid.

**Scheme 3:**
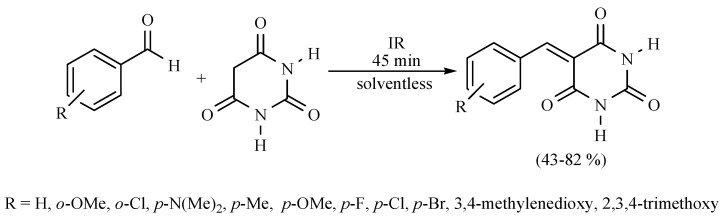
Synthesis of benzylidenebarbituric acids.

In this sense, the synthesis of benzylidenemalonates (Scheme 1) [9], benzylidenemalononitriles, benzylidenecyanoacetamides, benzylidenecyanoacetic acids (Scheme 2) [10], and benzylidenebarbituric acids (Scheme 3) [11] have been promoted in the presence of Tonsil Actisil FF (TAFF) [12], a bentonitic clay, as catalyst without the presence of solvent. In general, the reactions developed with good yields in short reaction times; these procedures involve simple work-up.

## 3. Nucleophilic Addition to Carbonylic Substrates

**Scheme 4:**
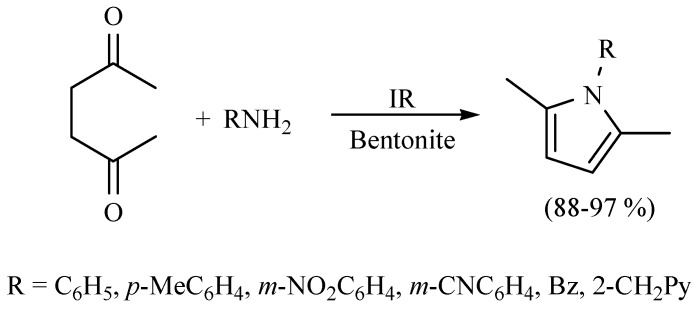
Production of *N*-substituted pyrroles.

The Paal–Knorr reaction is the most general synthetic method to prepare furans, tiophenes and pyrroles; according to this protocol, various *N*-substituted-pyrroles were obtained from primary amines and acetonylacetone using middle infrared irradiation as activating mode, and a natural clay as catalyst in the absence of solvent. The reaction proceeded with high yield in short reaction times (Scheme 4) [13].

**Scheme 5:**
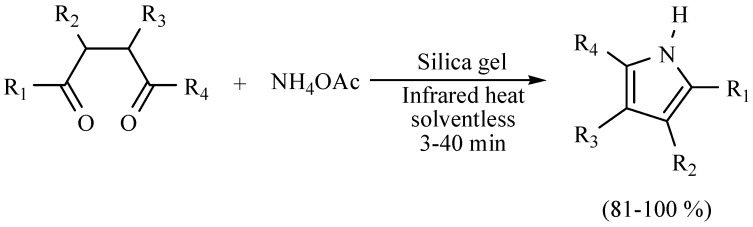
Synthesis of pyrroles.

Zhang and coworkers reported a pyrrole synthesis via a the Paal–Knorr protocol approach using inorganic ammonium salts as a nitrogen source and silica gel as the catalyst; the MIR activated reaction was conducted in a solid state, which means that no volatile organic compounds were used. The reaction time is short (Scheme 5) [14].

**Scheme 6:**
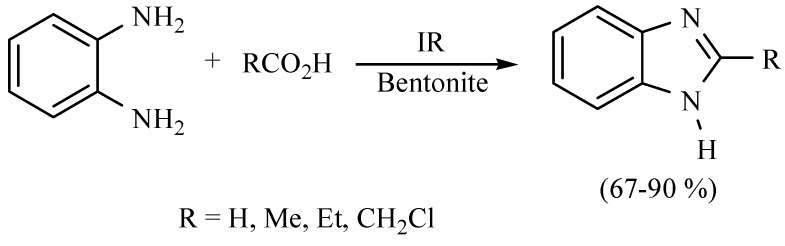
Obtention of benzimidazoles.

It is important to mention the benzimidazoles, a family of compounds with broad spectrum of pharmaceutical activity. In this sense, a green method has been developed using natural clay as the catalyst and employing MIR as the activating mode in solvent free conditions with good yield (Scheme 6) [15].

**Scheme 7:**
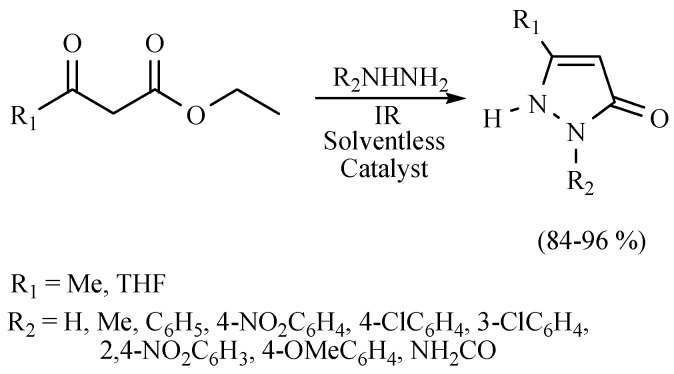
Production of pyrazolones.

Some pyrazolone derivatives have also been produced with excellent regioselectivity under MIR conditions and the catalytic activity of Co doped with ZnS nanoparticles. The catalyst enhances the overall capacity to absorb IR in the reaction mixture, providing the products with good to excellent yields (Scheme 7). The MIR activation was also compared to the conventional MH method [16].

**Scheme 8:**
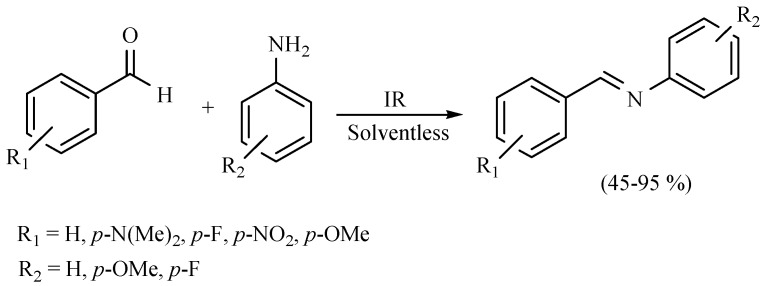
Formation of Schiff bases.

**Scheme 9:**
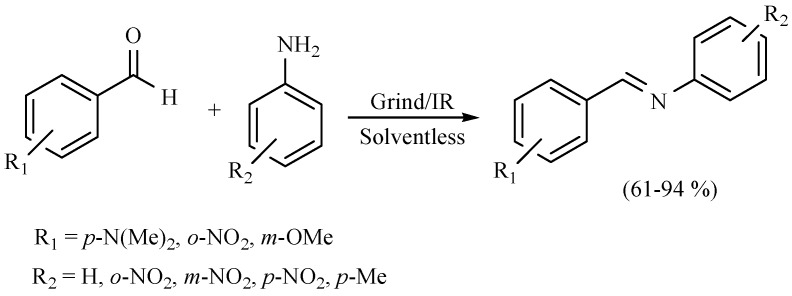
Production of Schiff bases.

Middle infrared irradiation has also been employed to promote the formation of a series of Schiff bases in the absence of solvent, with good yields. In general, if the substrate is a benzaldehyde bearing an electron-releasing group, the conversion rate decreases. In other words, when the substrate bears an electron-withdrawing group, a more efficient conversion took place. Moreover, an electron-releasing group on the aniline ring improves the yields (Scheme 8 and Scheme 9) [17,18].

**Scheme 10:**
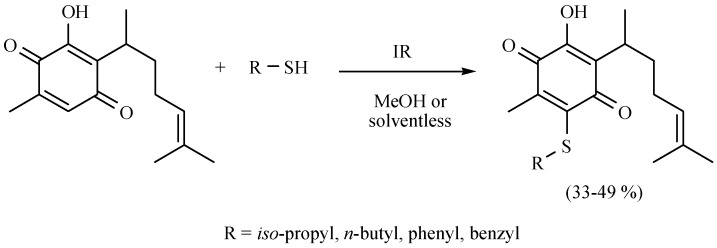
Obtention of sulfur-derivatives of perezone.

**Scheme 11:**
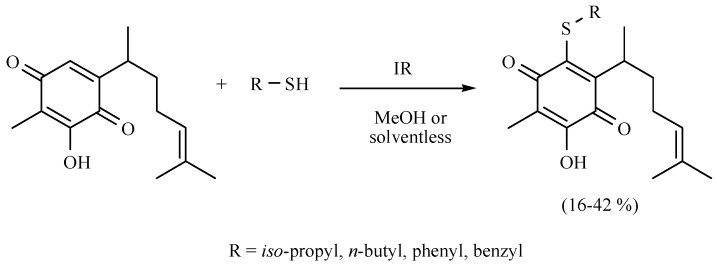
Obtention of sulfur-derivatives of isoperezone.

An excellent contribution to the chemistry of perezone, the first metabolite isolated in the New World, was performed by Martínez and coworkers [19]. In this regard, they reported a comparative study of perezone and isoperezone with a set of four different mercaptans: *i*-PropSH, *n*-BuSH, PhSH, and BzSH (Scheme 10 and Scheme 11, respectively). The transformations were conducted by 1,4-addition/oxidation tandem reaction using MIR as the activating mode, comparing solvent or solvent-free conditions, obtaining the new molecules with moderate yields.

## 4. Macrocycles.

**Scheme 12:**
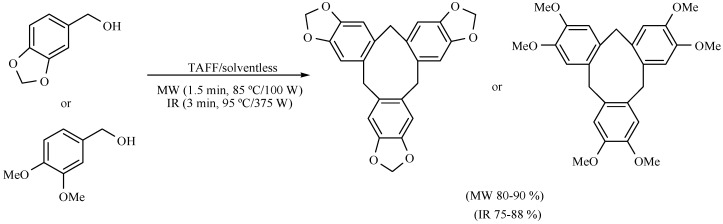
Formation of cycloveratrylene macrocycles.

**Scheme 13:**
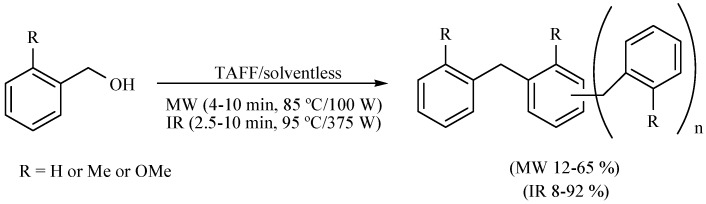
Production of benzyl oligomers.

The formation of cycloveratrylene macrocycles (Scheme 12) and benzyl oligomers (Scheme 13) from the corresponding benzyl alcohol has also been reported. The product generation was studied comparing two different activating modes: MW (85 °C/100 W, 1.50 min) and MIR (95 °C/375 W, 3–7 min), both in the absence of solvent, using TAFF as Lewis catalyst. The products were obtained in short reaction times with good yields in both cases. The molecules generated were the condensation products of the corresponding benzylic cations. These processes have the advantage that they eliminate both the use of solvents and halogenated starting materials. In addition, the natural clay is environmentally friendly in comparison to the typical mineral acid solutions or metallic catalysts. It is also offered as a time-efficient process [20].

## 5. Multicomponent Reactions

A key objective of Green Chemistry is to accomplish a reaction employing eco-conditions [1]; under such protocol, an ideal synthesis would be that by which a target molecule is produced quantitatively in one step from available and inexpensive starting compounds in an environmentally sustainable process. In this regard, multicomponent reactions (MCR), a significant subclass of tandem reactions [21], are considered as favorable green chemistry procedures, because at least three or more components react directly to yield a unique product, incorporating the atoms of the starting materials with great atomic efficiency; in other words, it is not necessary to isolate the reaction intermediates, making the complete procedure sustainable, simplifying the purification procedure and consequently enhancing the corresponding synthetic efficiency [22].

**Scheme 14:**
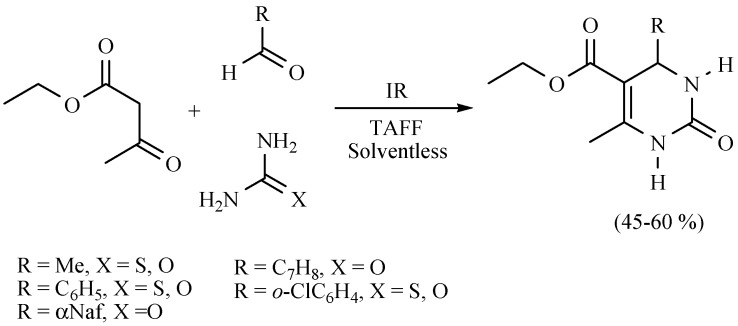
Synthesis of Biginelli esters.

**Scheme 15:**
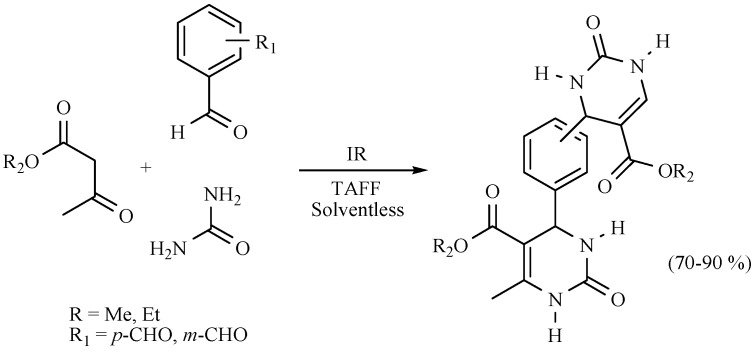
Production of *bis*-DHPMs.

The Biginelli (B-3MCR) and the Hantzsch (H-4MCR) procedures are common protocols for producing dihydropirimidinones (DHPMs) and dihydropyridines (DHPs), also known as Biginelli and Hantzsch esters, respectively. Several DHPMs were produced using MIR for the promotion of the reaction, with TAFF as catalyst under solvent-free conditions (Scheme 14) [23]. It is also worth mentioning that some *bis*-DHPMs (Scheme 15) have shown smooth muscle relaxation effects *in vitro* [24].

**Scheme 16:**
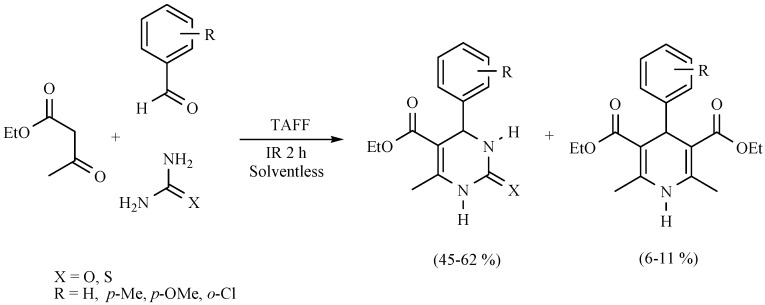
Comparative formation between DHPMs and DHPs.

It is also worth noting that competition between DHPs and DHPMs has been detected using the Biginelli protocol: the expected dihydropirimidinones were the main products, while the production of DHPs was promoted by the ammonia produced by decomposition of urea or thiourea employed as reagents (Scheme 16) [25].

**Scheme 17:**
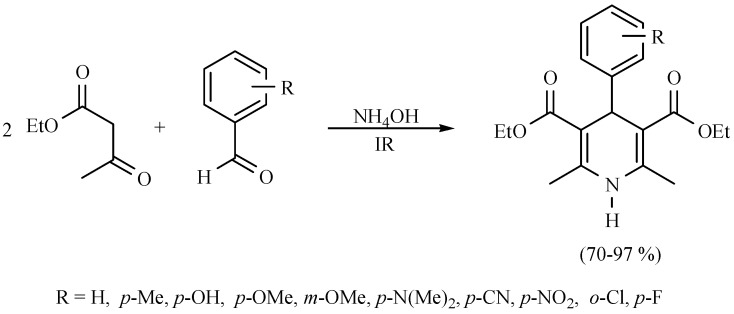
Synthesis of DHPs in water-based biphasic medium.

**Scheme 18:**
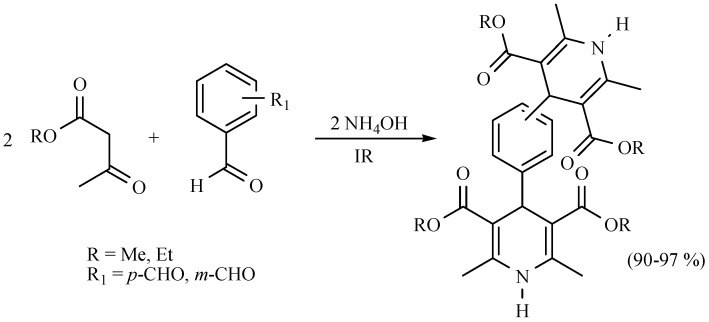
Formation of *bis*-DHPs.

Gómez-Pliego and coworkers [26] have also developed a procedure for the production of DHPs in a water-based biphasic medium (Scheme 17); in this research, a set of four *bis*-1,4-DHPs (Scheme 18) was also generated. It is important to highlight that these *bis*-1,4-DHPs exhibited vasodilatory and anticonvulsant effects [27,28].

**Scheme 19:**
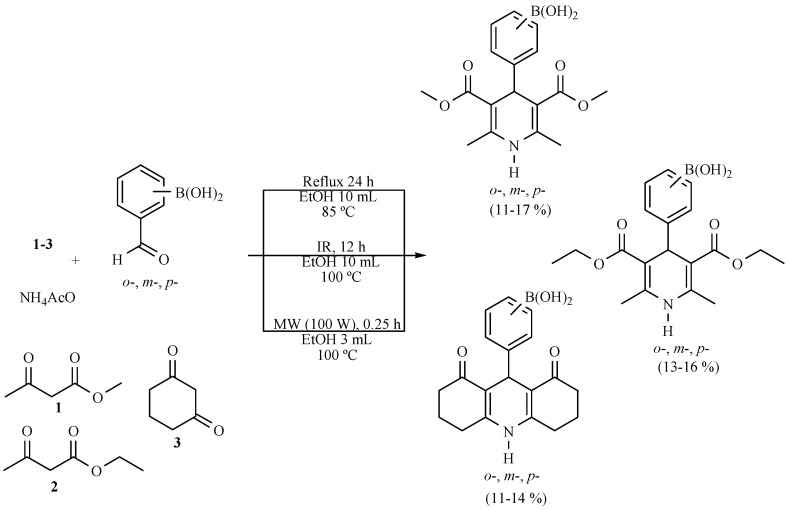
Production of boron-DHPs.

**Scheme 20:**
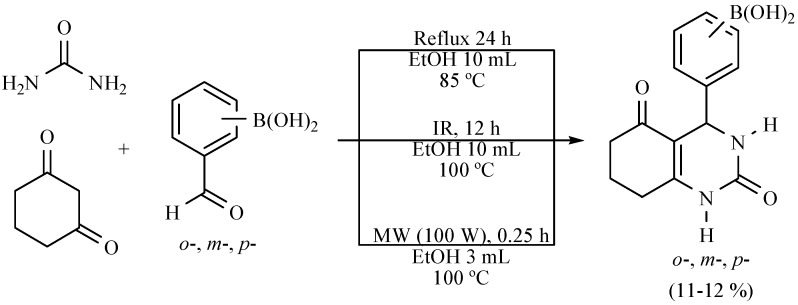
Formation of boron-DHPMs.

Several new hybrid-boron-containing 1,4-dihydropyridines (Scheme 19) and 3,4-dihydropyrimidinones (Scheme 20) were obtained from the corresponding regioisomeric formylphenylboronic acids. The target molecules were obtained in a comparative study (MW *vs*. MH *vs*. NIR) using ethanol as a green solvent. The infrared irradiation process was not the best procedure, but it can be considered as an alternative for generating these molecules according to the green chemistry protocol [29].

**Scheme 21:**
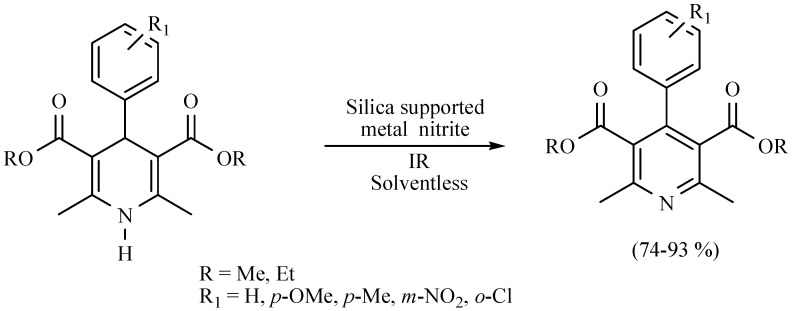
Oxidation of DHPs.

The oxidation of DHPs has been conducted using catalytic amounts of silica-supported transition metal nitrates such as Ni(NO_3_)_2_·6H_2_O and Co(NO_3_)_2_·6H_2_O under solvent-free conditions, previously grinding in a mortar (Scheme 21) [30].

**Scheme 22:**
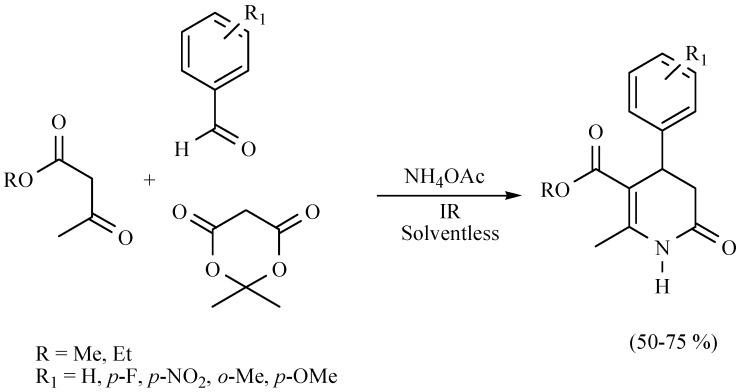
Multicomponent formation of DHPDs.

**Scheme 23:**
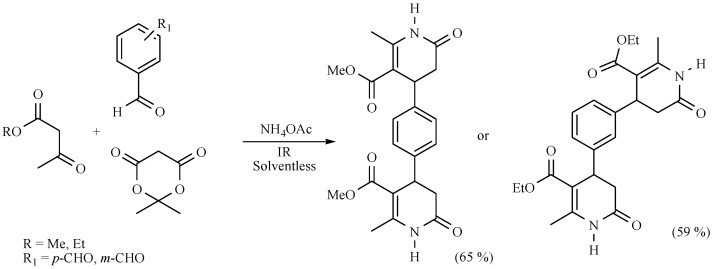
Production of *bis*-DHPDs.

In a research developed by Noguez and coworkers to produce dihydropyridinones (DHPDs) in a M-4MCR protocol in the absence of solvent, NIR was employed as the activating mode with better yields (50%–75%) in comparison to MH (40% or less) (Scheme 22). Two *bis*-3,4-DHPDs were also reported (Scheme 23) [31].

**Scheme 24:**
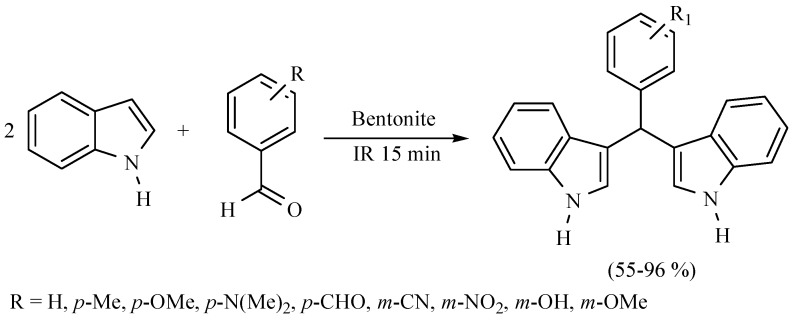
Obtention of diindolylmethanes.

Diindolylmethanes are a very important class of chemical compounds; many of them show preventive antineoplasic activity. Consequently, novel procedures, mainly under the green chemistry protocol, are welcome. In this sense, several diindolylmethanes were produced from indole and various aryl aldehydes, using MIR to activate the reaction in presence of bentonitic clay as catalyst (Scheme 24) [32]; some of these molecules exhibited antitumor activity *in vitro* against murine L5178Y lymphoma cells [33].

**Scheme 25:**
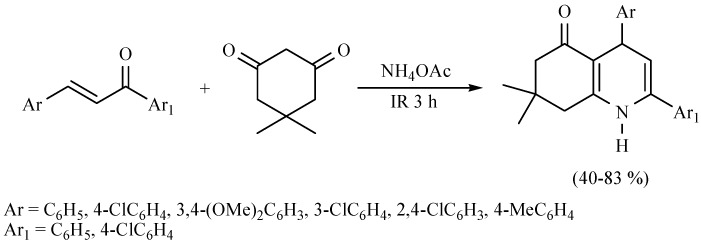
Production of octahydroquinolines.

The reaction to produce various octahydroquinolines using infrared irradiation for 3 h in the absence of solvent has also been performed between an active methylene dione with various diarylpropenones in the presence of ammonium acetate. The reactions proceeded with good chemical yields. It is worth mentioning that the presence of electron-withdrawing or electron-donating groups are not important to the corresponding yields (Scheme 25) [34].

**Scheme 26:**
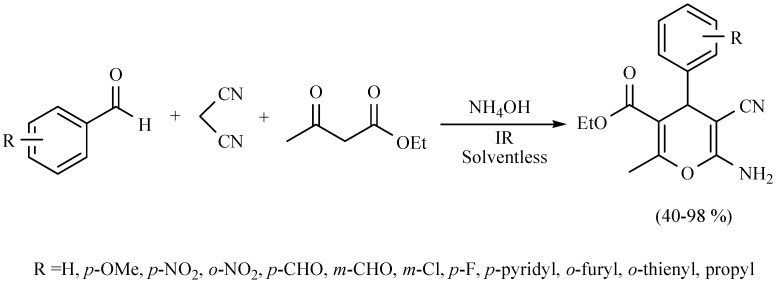
Multicomponent synthesis of 4*H*-pyrans.

Molecules of the class 4*H*-pyrans are very significant because their basic portion is present in a great diversity of secondary metabolites and several biologically active products. In this sense, a novel and efficient three-component method for the synthesis of 4*H*-pyrans derivatives was developed. It was catalyzed with NH_4_OH and promoted by infrared irradiation. The offered method involves a multicomponent reaction in short reaction times under eco-friendly conditions (Scheme 26) [35].

**Scheme 27:**
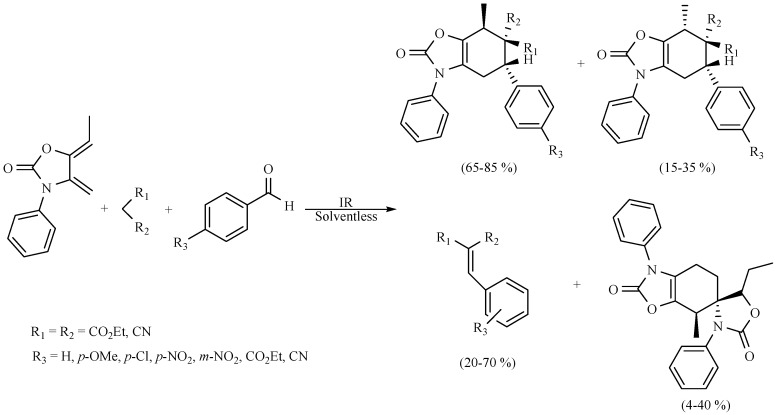
Obtention of tetrahydrobenzo[*d*]oxazol-2-ones.

A one-pot three-component process was developed to obtain several tetrahydrobenzo[*d*]oxazol-2-ones using MIR in order to activate the reaction under solvent-free conditions (Scheme 27) favoring the *para*-*endo* cycloadducts with respect to the *meta* or *para*-*exo* adducts [36]. No catalyst was required.

**Scheme 28:**
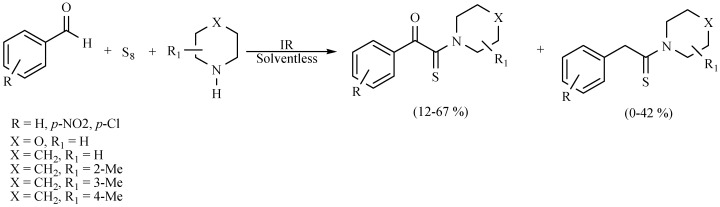
Production of thioamides and α-ketothioamides.

A novel mode for the production of thioamides and α-ketothioamides has also been reported. It is an appropriate modification of the Willgerodt–Kindler reaction: solvent-free, non-catalyst and IR energy as activating mode of reaction. The results showed that the α-ketothioamides is the main product and the corresponding Willgerodt–Kindler is the minor product. These results show better yields in comparison to previously reported procedures (Scheme 28) [37].

**Scheme 29:**
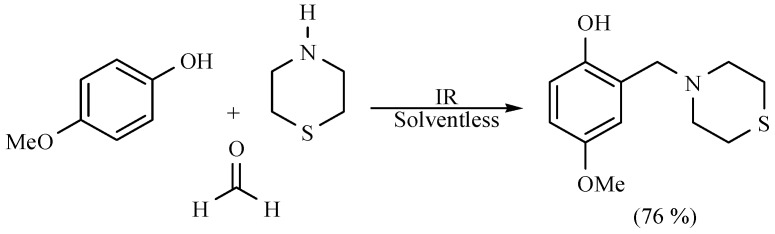
Formation of nitrogen containing compounds.

Some other interesting nitrogen containing compounds have been reported, obtained by means of a one pot procedure. Among them are several derivates of piperidine, morpholine and thiomorpholine; the target compounds were obtained with good yields in the absence of solvent using MIR as activating source with short reaction times (Scheme 29) [38,39].

## 6. Reduction–Oxidation Reactions

Reduction of several functionalities represents a corner-stone reaction within organic chemistry. Owing to its importance, a great deal of effort has been dedicated to the development of green catalytic methods for the corresponding reductions. On the other hand, the oxidative practice involves important steps for the inter-conversion of chemical functionalities from readily available starting materials. Despite its importance, traditional oxidative methodology has some disadvantages, including the use of environmentally incompatible reagents (such as chromium, nickel, or copper-based reagents) and the potential for undesired side reactions [40]. Thus, in relation to the green chemistry protocol, photo-catalysis is an environmentally friendly technique to eliminate toxic organic substances in air and water [41].

The production of reduced graphene oxide (GO) is important because of its employment as sensors, and among other applications. To this point, Guo and coworkers presented an alternative for the efficient photothermic reduction of GO by MIR, producing both porous reduced GO and crack-free highly conductive thin films of reduced GO. The authors determined the reduction of GO by FT-IR in addition to other studies, including thermogravimetric analysis, Raman, and X-ray photoelectron spectroscopy. This work is a good green method because no chemical reducing agent was employed [42].

**Scheme 30:**
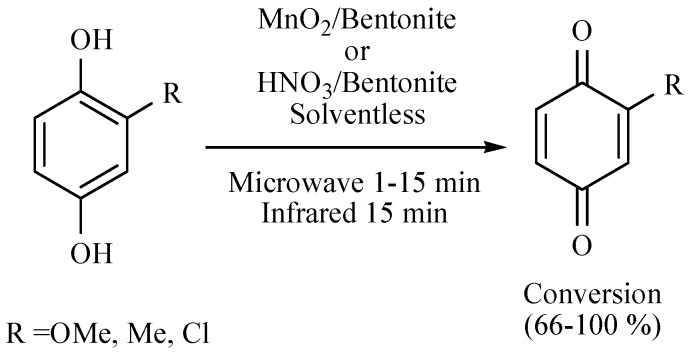
Oxidation of hydroquinones to quinones.

An example of an oxidation assisted by MIR, is the conversion of hydroquinones to quinones reported by a simple and straightforward procedure employing MnO_2_ or HNO_3_, both separately supported on a bentonitic earth and in solvent-free conditions (Scheme 30) [43].

**Scheme 31:**
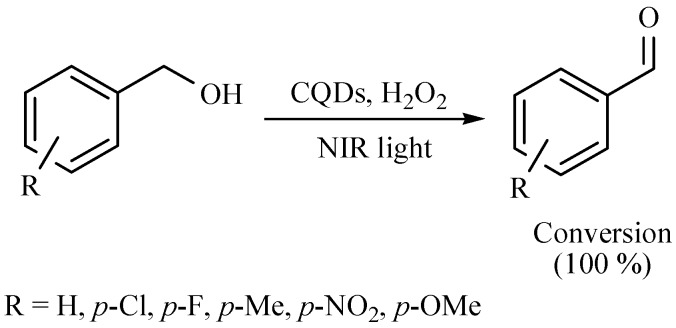
Obtention of benzaldehydes from alcohols oxidation.

The selective oxidation of alcohols is a fundamental and significant transformation for the production of fine chemicals; in this sense, the ultraviolet and visible light driven photocatalytic systems for alcohol oxidation have been previously reported. In this work, the authors informed that the named carbon quantum dots (CQDs) are able to behave as valuable NIR form photocatalyst for the selective oxidation of several benzylic alcohols to their corresponding benzaldehydes with good yields. Based on its NIR driven photo-induced electron transfer property and its photocatalytic activity for H_2_O_2_ decomposition, this metal-free catalyst performed the oxidations (Scheme 31) [44].

An oxidative photocatalytic decomposition of 2,4-dichlorophenol in aqueous solution was reported using Cu_2_(OH)PO_4_ microcrystals under NIR; the authors explain the photocatalytic activity of Cu_2_(OH)PO_4_ by an effective separation of the electron hole pairs from CuO_4_(OH) to CuO_4_(OH)_2_ octahedral [45].

Ikeue and coworkers have reported the photo-oxidation of 1,3-diphenylisobenzofuran in aerated toluene employing the annulated dinuclear palladium (II) phthalocyanine complex as an effective photo-oxidation catalyst using NIR as activating reaction mode [46].

## 7. Miscellaneous Reactions

**Scheme 32:**
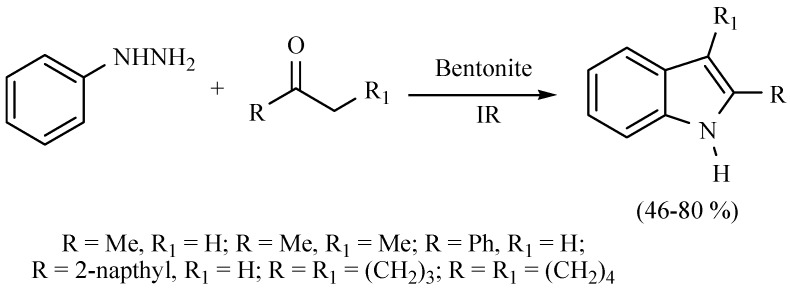
Production of indoles.

A Classical mode to produce indole is the Fischer method; in this regard, MIR was used as the activating mode in a modified procedure to produce related compounds, employing TAFF as a Lewis acid catalyst, with good reaction yields (Scheme 32) [47].

**Scheme 33:**
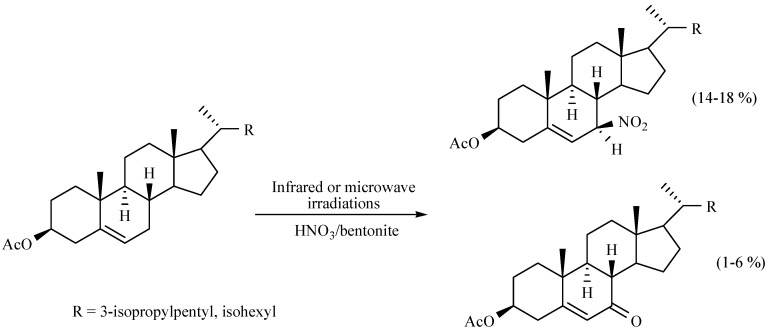
Preparation of nitro steroids.

A method to prepare nitro steroids was reported by Jiménez-Estrada and coworkers. In this study, 7α-nitro-3β-sitosterol and 7α-nitro-3β-cholesterol acetates were produced using the HNO_3_/bentonite system. The corresponding reaction mixtures were comparatively activated with MIR or MW over 45 and 27 min, respectively (Scheme 33). The title compounds were the most abundant products. The authors suggest a mechanism involving the possible formation of NO_2_ free radicals [48].

**Scheme 34:**
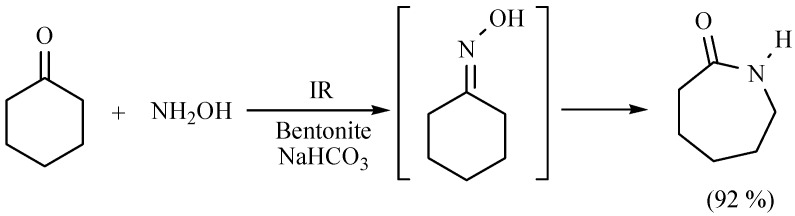
Obtention of ε-caprolactam.

The production of ε-caprolactam, the required substrate to obtain nylon-6, was performed in a comparative study, involving a modified Beckmann rearrangement protocol. The compared activating modes were MW, MIR, MH and US. Bentonitic clay was employed as the catalyst. It is important to note that the MIR procedure offered the best yields (Scheme 34) [49].

Among self-activating reactions, the peptide formation reaction is the most interesting one. In this sense, when *N*-phosphoamino acid (DIPP-aa_1_) and an amino acid (aa_2_) were dissolved in aqueous solution and irradiated by infrared light, the corresponding oligopeptides were formed. The authors propose that the oligopeptides might be formed in the self-activating reaction process via intramolecular carboxylic–phosphoric–anhydride intermediate. In addition, is noteworthy that no matter what kind of entities were used, there were only two types of produced peptides: DIPP-(aa_1_)*_n_* or DIPP-(aa_1_)*_n_*_-1_-aa_2_ [50].

The production of several alpha-alumina powders was described by Hernández and González following the conventional Pechini synthesis in order to study its dielectric behavior. The corresponding polymerization was conducted between nitrate or lactate aluminum and aqueous citric solution and ethylene glycol, employing MW or MIR as comparative activating sources. However, the best final alumina powder was synthesized by applying MW [51].

The formation of SnO_2_ nanoparticles with four different sizes was performed using infrared irradiation and typical thermal treatment. The title materials have considerable value in gas sensors and dye-based solar cells. The authors reported that the best size of this type material is ~11 nm because it has fewer lattice defects in addition to suffering less electrochemical agglomeration [52].

Different materials are used as protective coatings on metallic substrates because of their interesting properties, including high thermal conductivity, low electric conductivity, and excellent thermal stability. In this sense, boron nitride (BN) in a hexagonal form was prepared using a liquid polyborazylene as a BN source and it annealing was activated by infrared irradiation to perform the polymer-to-ceramic conversion. It is the first report about the preparation of BN coating on titanium substrate with liquid BN polymeric precursor through MIR; this study creates an access toward the molecular design of hexagonal boron nitride coatings as protective materials for low melting point metals [53].

Is well known that the percentage of UV light in the solar spectrum is only 5%, which is very low compared to visible light (~48%) and near-infrared light (~44%). The low usage of sunlight has restrained photocatalytic efficiency to pure TiO_2_, as the most promising photocatalyst, for environmental remediation. With this in mind, a photocatalyst consisting of YF_3_:Yb^3+^ and TM^3+^/TiO_2_ core/shell nanoparticles has been reported demonstrating that NIR energy can be used as an activating source. Complementarily, methylene blue was used in this study, which suffers decomposition by core/shell nanoparticles under a 980 nm laser and NIR [54].

**Scheme 35:**
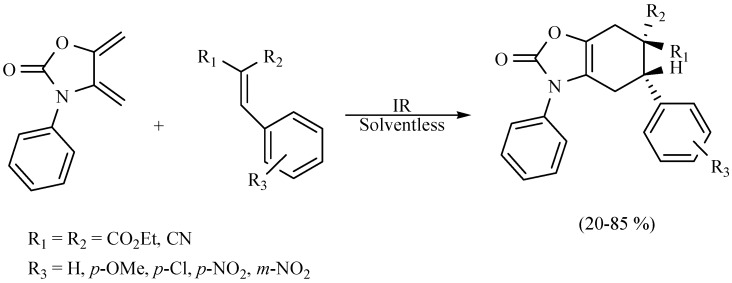
Synthesis of 2-oxazolidinone derivatives.

A new, efficient, regio- and stereoselective Diels–Alder reaction (Scheme 35) was developed by Flores-Conde and coworkers, between a series of Knoevenagel adducts as dienophiles in the presence of *exo*-2-oxazolidinone with MIR as activating source under solvent-free conditions and in the absence of a catalyst [36].

**Scheme 36:**
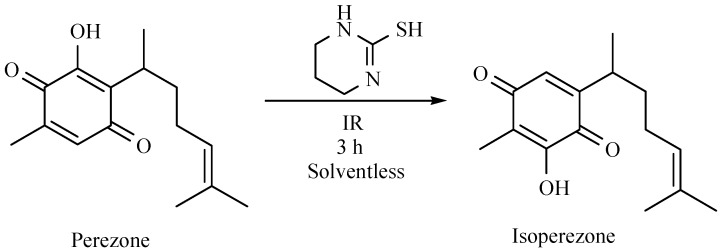
Production of isoperezone.

It is worth nothing that isoperezone was produced using perezone as substrate in the presence of 3,4,5,6-tetrahydro-2-pirimidinethiol and in absence of solvent using MIR as activating source. The process was carried out over three hours and the reaction yield was similar to the previously reported procedure, typical thermal conditions for 12 h (Scheme 36) [19].

**Scheme 37:**
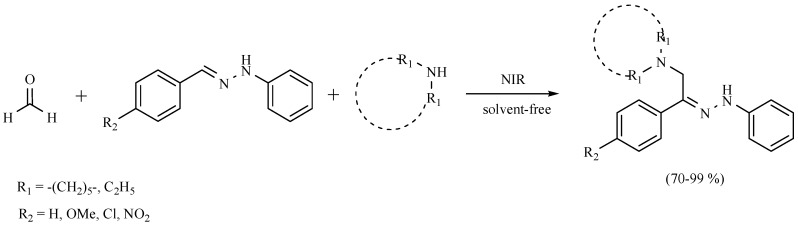
Synthesis of amino(aryl)phenylhydrazones.

**Scheme 38:**
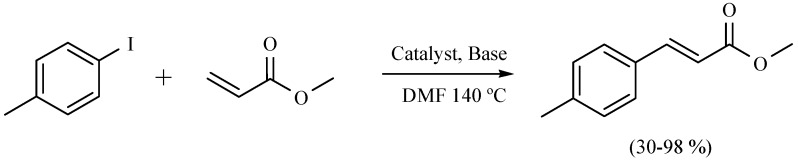
Formation of C-C bond by mean Heck coupling.

Recently, the Mannich coupling reaction between various arylhydrazones, formaldehyde and a secondary amine to generate the corresponding (*Z*)-(aminomethyl(aryl)phenylhydrazones assisted by near infrared irradiation under solvent-free conditions has been performed (Scheme 37). In addition, the catalytic potential of the obtained molecules in the palladium-catalyzed NIR-assisted Heck coupling reaction was evaluated. The coupling products were obtained in high yields and in short reaction times (Scheme 38) [55].

**Scheme 39:**
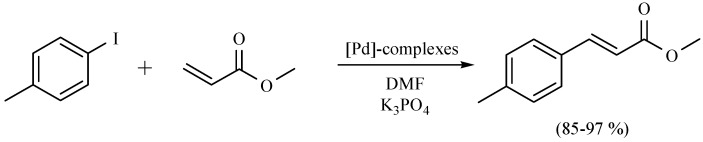
Production of C-C bond by mean Mizoroki-Heck cross-coupling.

In another recent report, an alternative and environmentally friendly strategy to promote the Mizoroki–Heck cross-coupling reaction using NIR as the activating mode of reaction and employing palladacycles as precatalyst has been offered. A comparison with the classical use of reflux conditions, and commercial sources of palladium complexes shows the advantages of this new alternative for promoting the target reactions. The authors highlight this report as the first time NIR was used for this purpose. The results obtained indicate that NIR can be considered an excellent, economical and accessible alternative to promote this coupling reaction, showing advantages such as short reaction times and good yields, facilitating access to a clean, simple and economic methodology comparable to those involving microwaves (Scheme 39) [7].

As was mentioned at the beginning of the review, the infrared spectrum is divided into three zones (near, middle, and far).With this in mind, it is also worth mentioning that the majority of chemical reactions described herein have been made using middle infrared irradiation. In this sense, Noguez Cordova and coworkers performed a comparative study between NIR and MIR [56].Thus, the results of various different reactions are summarized in Table 1. During the development of this work, the expected molecules were obtained using NIR compared with MIR as the activating mode. As can be seen, the reaction times of the NIR mode in comparison to the MIR procedure are significantly reduced (to half and in some cases even to a third). Other advantages mentioned by the authors include: the immediate response time for the heat source (≤1 s), the efficient use of applied energy by convection, and the longer life time of the tungsten-halogen filament. In addition, in comparison to the typical mantle heating mode, the reactions are “greener”, offering an environmentally friendly way of practicing chemistry.

## 8. Novel Results from Our Research Laboratory

The results displayed in this section correspond to recent, novel results obtained by our research group. It is important to note that they are currently being written up or have recently been submitted for publication.

**Scheme 40:**
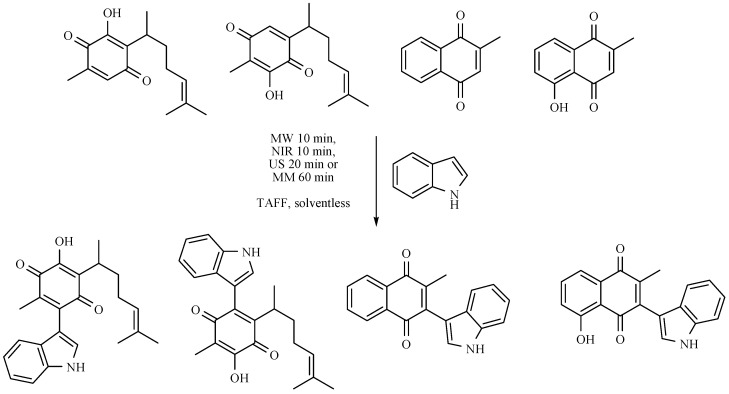
Production of several indol-derivatives of natural quinones.

Several indol-derivatives from four natural quinones (perezone, isoperezone, menadione and plumbagine) have been prepared, employing four non-conventional activating modes, including NIR, under solvent-free conditions and with the employment of TAFF as catalyst (Scheme 40). The target molecules are under several pharmacological assays.

**Scheme 41:**
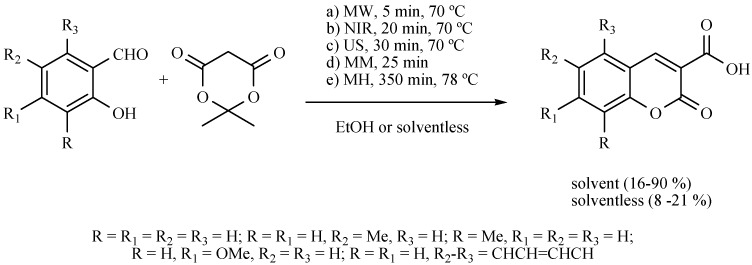
Formation of coumarins.

Various coumarins have also been produced to compare several non-conventional activating sources with the typical mantle heating, in the presence or absence of ethanol as solvent and without catalyst (Scheme 41). The reactions proceeded in short reaction times with moderate to good yields. It is worth mentioning that NIR has shown the best yields.

**Figure 1:**
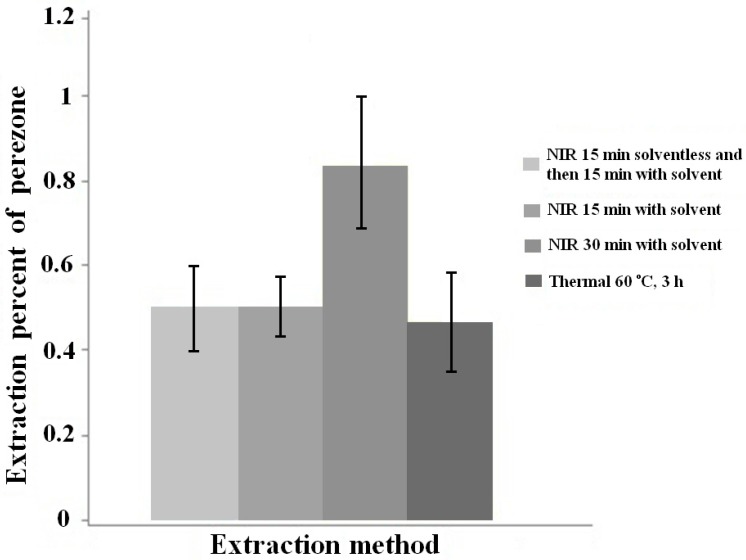
Comparison of extractions percent between NIR and MH methods.

Related to natural products extraction, a wide study about the extraction of perezone from roots of *Acourtia* plants has recently been performed by our group to compare various modes: MH, NIR, MW, US and supercritical CO_2_. It is worth mentioning that the yields obtained from the extraction using NIR for 15 min were statically equal to the conventional thermal extraction (reflux for 3 h). Figure 1 exhibits a comparative histogram between MH and NIR extractions.

**Figure 2:**
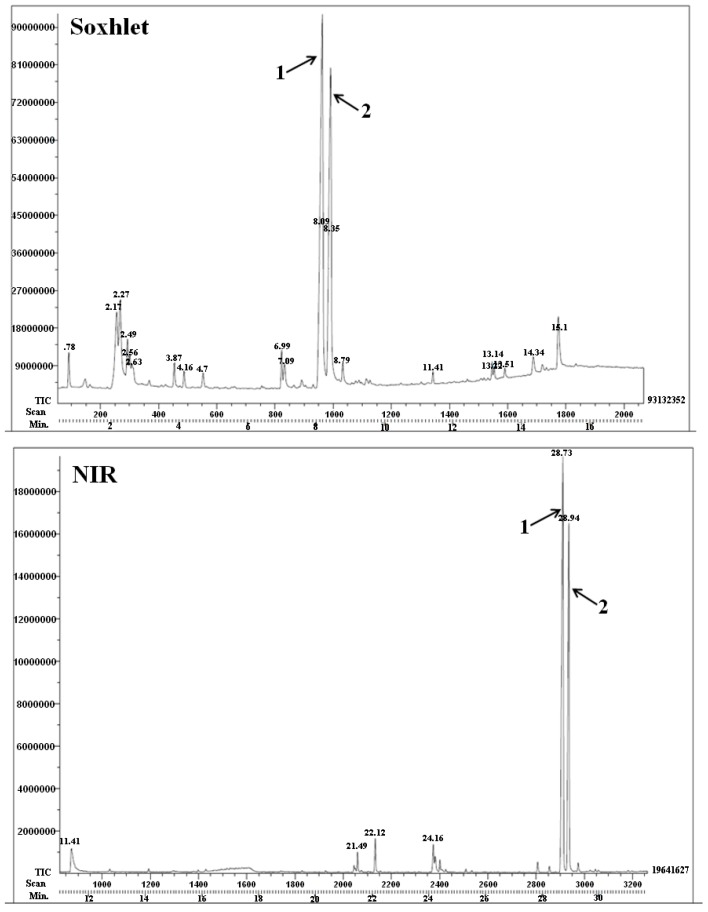
Chromatograms extraction of capsaicin (**1**) and dihydrocapsaicin (**2**).

The extraction of capsaicin and dihydrocapsaicin from habanero pepper powder has been evaluated recently to compare several modes: MH-Soxhlet, NIR, MW, and US using ethanol as the solvent. The best results were: 30 min for NIR, 6 min for MW and 65 min for US. As an example, the chromatograms of Soxhlet and NIR processes are displayed (Figure 2).

## 9. Natural Products Extractions

As is well known, several methods are available to extract the metabolic compounds present in vegetable species: solvent [58], pressurized hot water [59], ultrasound [60], microwave [61,62,63] and supercritical fluids [64,65]. However, it is worth noting that infrared irradiation, mainly FIR, has also been employed. Thus, we have summarized the corresponding results from a literature searching Table 2. As can be seen, it contains the studied vegetable material, a brief description of the corresponding procedure, the present metabolites and the respective reference.

## 10. Conclusions

In this review, a deep literature search, appropriate analysis and adequate organization of the obtained information, present the use of infrared irradiation (in its three zones, NIR, MIR, and FIR) as a clean and effective mode to activate a reaction, as well as an appropriate procedure for the extraction of natural products from corresponding vegetable materials. In addition, taking into account many of the offered reaction conditions (solvent-free and catalysis, for example) in the examined papers, infrared irradiation is shown to be a good green approach.

## Figures and Tables

**Table 1:** Near *vs.* middle infrared irradiation for the completion of organic reactions.

Substrate	Reactions		MIR: Time/Temp	NIR: Time/Temp
	Reagent	Product	Literature [57]	This Work [56]
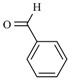	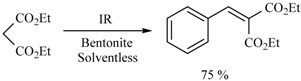		15 min/80 °C	7 min/170 °C
	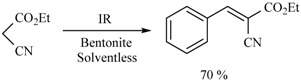		15 min/80 °C	7 min/170 °C
	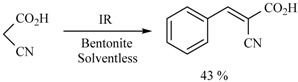		15 min/80 °C	7 min/170 °C
	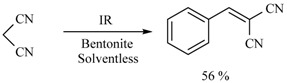		15 min/80 °C	7 min/170 °C
	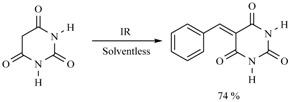		45 min/80 °C	20 min/170 °C
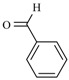	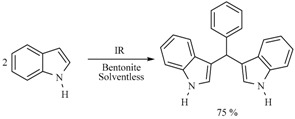		15 min/80 °C	7 min/170 °C
	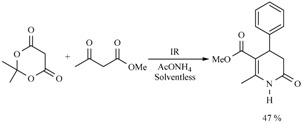		3 h/80 °C	50 min/170 °C
	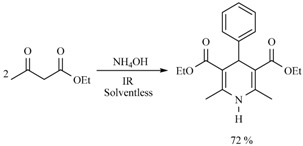		3 h/80 °C	50 min/170 °C
	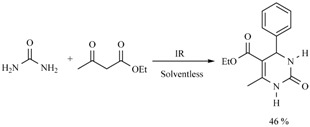		3 h/80 °C	1.5 h/170 °C
	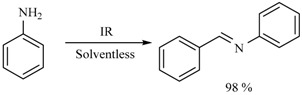		40 min/80 °C	20 min/170 °C

**Table 2:** Natural products extractions by infrared irradiation.

Vegetable Material	Work Description	Compounds Analyzed	Refs.
*Oriza sativa* L*.* (Rice cultivar)	The rice hulls were treated with FIR for 30 min. After irradiation, a methanolic extract was prepared at room temperature for 1 h. The yields and antioxidant activity of the extract was better in comparison to the conventional method. In addition, The obtained results indicate that FIR could liberate and activate covalently bound phenolic compounds with antioxidant activities.	*p*-Coumaric acid; 3-vinyl-1-oxybenzene; 4-hydroxybenzaldehyde; vanillin; 4-hydroxybenzoic acid; and 4,7-dihydroxyvanillic acid.	[66]
	Rice hulls pulverized and passed through a 48-mesh molecular sieve were treated with FIR for 2 h, and then a methanolic extract was prepared at room temperature overnight. The antioxidant effect of far infrared-treated rice hull (FRH) extracts in irradiated turkey breast meat was compared with that of sesamol and rosemary oleoresin. The FRH extracts significantly decreased thiobarbituric acid-reactive substances values and volatile aldehydes (hexanal, pentanal, and propanal) and was effective in reducing the production of dimethyl disulfide responsible for irradiation off-odor in irradiated raw and cooked turkey meat during aerobic storage.		[67]
*Sesamum indicum* L. (Sesame)	Sesame seeds were treated with FIR. The sample was defatted and the residue was used to prepare a methanolic extract by shaking overnight at room temperature. After that, the corresponding antioxidant activities were determined.	*p*-Hydroxybenzoic acid; *o*-coumaric acid, vanillic acid; *p*-coumaric acid, isoferulic acid; sesamol and tocopherol.	[68]
*Arachis hypogaea* L. (Peanut)	Peanut hulls dried under room temperature, were treated with FIR for 5 to 60 min at 150 °C. The irradiated material was extracted with distilled water at room temperature overnight. The total phenol contents, radical scavenging activities, and reducing power of these water extracts were determined.	2-Methoxyphenol; 2-hydroxy-4-methoxybenzoic acid; 2-methoxy-2-vinylphenol; 4-hydroxy-3-methoxybenzaldehyde; 2,4-*bis* (1,1-dimethylethyl)phenol; 4-hydroxy-3-methoxybenzenacetic acid; vanillyl alcohol; methylcinnamate.	[69]
*Camellia sinensis* var. sinensis (Green tea leaves)	In a first instance, the processed green tea leaves were treated with FIR at 80–150 °C for 10 min. Then, the leaves were prepared by soaking them in boiling water and the corresponding physicochemical characteristics were determined. The applied FIR at 90 °C increased total phenol and total flavanol contents in comparison with non-irradiated control. FIR also significantly affected the amounts of epigallocatechin and epigallocatechin gallate. These results support the idea that FIR could be a useful method for increasing the health-promoting properties of green tea.	Polyphenolic compounds; caffeine; (−)-epicatechin; (−)-catechin; (−)-epicatechingallate; (−)-catechingallate; (−)-epigallocatechin; (−)-gallocatechin; (−)-epigallocatechingallate; (−)-gallocatechingallate; and ascorbic acid.	[70]
	Green tea leaves were processed by seven modes, six of them using FIR at 90 °C for 10 min at different moments of the manufacturing process (during roasting and drying and finally on the dried product). The physicochemical characteristics of the green tea were determined. The obtained results indicate that the chemical properties of green tea are significantly affected by FIR at specific stages of the manufacturing process of green tea leaves, providing a high-quality green tea.		[71]
	The effect of FIR on the physicochemical characteristic of green tea during processing was determined. Green tea leaves were irradiated by FIR during the roasting and drying step; then, the leaves were extracted by soaking them in water, and the components analyzed. The obtained results show that FIR, during the green tea process, affected its chemical properties. In addition, the authors comment the FIR can be employed to increase the physicochemical qualities of green tea.		[72]
*Puerariae radix*	The antioxidant activity modulated by FIR on *Puerariae radix* extract was evaluated. In addition, it is mentioned that this specimen has been widely used in Eastern Asia to treat the common cold and influenza and as an antidipsotropic agent. FIR at 110 °C for 40 min increased the values of total phenol contents, radical scavenging activity, reducing power and puerarin content in the corresponding methanolic extract in comparison to the non-irradiated material. The obtained results suggest that FIR could be used to increase the quality of the studied herb.	Polyphenols compounds, isoflavonoids such: puerarin, daidzein, daidzin, luteolin and biochanin A.	[73]
*Morus alba* L. (Mulberry)	The antioxidant and physical properties, and consequently the change in polyphenolic compound in mulberry tea, as influenced by FIR, were determined. This study aimed to use FIR for convection drying to improve physical quality and antioxidant properties of mulberry tea. In addition, it was demonstrated that the intensity of FIR affected the total phenolic and flavonoid contents.	*p*-Coumaric acid; benzoic acid; (+)-catechin; chlorogenic acid; vanillic acid; syringic acid; sinapic acid; protocatechuic acid; ferulic acid; gallic acid and caffeic acid.	[74]
*Radix Salviae miltiorrhizae* (Dashen)	In this study, FIR was employed to extract the active compounds from *Radix Salviae miltiorrhizae*; the optimal conditions were as follows: extraction time 15 min; extraction solvent 70% *v*/*v* methanol in water solution; and solid/liquid ratio of 0.1:15 (g/mL). Thus, four phenolic compounds and four diterpenoids were isolated. It is also worth noting that FIR was compared with conventional heat-reflux extraction, ultrasound-assisted extraction and microwave-assisted extraction. The FIR mode resulted as the best mode to extract the target molecules.	Danshensu; protocatechuic acid; protocatechuic aldehyde; salvianolic acid B; dihydrotanshinone; cryptotanshinone; tanshinone I; and tanshinone II A.	[75]
*Lycium barbarum* Linn	FIR-assisted methanolic extraction was employed to extract rutine, quercetin and gentisic acid. The effect of detection potential, irradiation time, and the voltage applied, was investigated in order to acquire the optimum analysis conditions. It is worth noting the use of capillary electrophoresis as a complementary method for the determination of bioactive constituents. The yields were similar to the conventional methods but at lower time.	Quercetin; rutin; and gentisic acid.	[76]
*Glycyrrhiza uralensis* Fisch (Licorice roots)	Licorice roots powders were treated with FIR for 30 min at 120–200 °C. After irradiation, a 50% ethanol extract was prepared at room temperature for 1 h. The total phenolic and flavanol contents in addition to the radical scavenging activities were determined. The contents of liquiritin, glycyrrhetic acid and glycyrrhzin increased.	Liquiritin; glycyrrhetic acid; and glycyrrhzin.	[77]
*Lysium chinensis* Mill	Folium Lysium Chinensis, a commonly used traditional Chinese medicine, is the dried leaf of *Lysium chinensis* Mill, and it has been employed to treat consumptive disease, fever, polydipsia, headache, metrorrhagia, and carbuncle. In this work, a method based on capillary electrophoresis and FIR-water assisted extraction has been developed to determine mannitol, sucrose, glucose and fructose quantities. The extraction time was significantly reduced to 7 min compared with several hours for the conventional hot solvent extraction.	Mannitol; sucrose; glucose and fructose.	[78]
Grape seeds	In this work, FIR-assisted extraction was combined with HPLC for the determination of catechin, epicatechin and procyanidin B2 in grape seeds. The grape seeds powder was dispersed in different mixtures of water–methanol. These mixtures were irradiated for 30 min. The extraction efficiency was compared with other methods, including microwave extraction, ultrasonic extraction and the classical mantle heating.	Catechin; epicatechin; and procyanidin B2.	[79]
*Rhododendron mucronulatum* Turcz	A combined method with FIR procedure was employed for the extraction of two flavones and three phenolic acids from the dried leaves of *Rhododendron mucronulatum* Turcz, commonly used in traditional Chinese medicine. The dried and pulverized leaves were dispersed in 60% aqueous methanol and treated with FIR for 6 min under reflux with methanol. The results indicate that FIR significantly enhances the extraction efficiency and reduces the time from 3 h to 6 min in comparison with the conventional heat solvent extraction.	Rutin; farrerol; syringic acid; vanillic acid and 4-hydroxybenzoic acid.	[80]
*Tagetes erecta* L. (marigold flower)	Marigold flower has long been used as a food colorant and ingredient in human food and animal feed. Drying is one the most important processes for the production of marigold powder. Therefore, in this work, the effects of different drying processes—freeze-drying, hot air drying and combined FIR-hot air convection—on the color, in addition to the carotenoids and phenolic compounds presence in the marigold flowers were evaluated. The obtained results demonstrate that FIR-hot air convection should be considered as a suitable drying method for marigold in order to preserve its color, antioxidant properties and bioactive compounds.	*p*-Coumaric acid; catechin; chlorogenic acid; vanillic acid; syringic acid; sinapic acid; protocatechuic; ferulic acid; gallic acid; caffeic acid and three carotenoids (lycopene, β-carotene and lutein).	[81]
*Picrorhiza scrophulariiflora* Pennell	The obtained results in this work show that FIR-assisted non-ionic surfactant extraction is good, efficient and a green analytical preparatory technique for the rapid extraction and pre-concentration of pharmacologically active ingredients from *Picrorhiza scrophulariiflora* Pennell.	Picroside I and picroside II.	[82]
*Hibiscus cannabinus* L. (Kenaf leaf tea)	In this work, the effect of FIR on the total polyphenol, total flavonoid, antioxidant activity and angiotensin I-converting enzyme inhibition ability were investigated in Kenaf leaf tea. The corresponding obtained data indicate that FIR at 60 °C increased the total polyphenol contents and flavonoid contents; in addition, the free radical scavenging and the lipid peroxidation inhibition activity were also increased.	Polyphenolic and flavonoid compounds; kaempferitrin, afzelin; α-rhamnoisorobin; and kaempferol.	[83]
*Glycine max* M. (black soy bean)	The goal of this work was to study the optimal temperature and time to obtain daidzein and genistein from their corresponding glycosides, employing FIR as a thermal source on black soybean. In other words, FIR is offered as a convenient green tool for the chemical conversion of isoflavone glycosides in soybean.	Daidzein and genistein.	[84]
*Fagopyrum* spp. (Buckwheat)	In this work, the FIR effect on total polyphenol, total flavonoid content, antioxidant properties and aglycone quercetin production in tartary buckwheat sprout were investigated. The powder of the herbal material was mixed with water and exposed to FIR at different temperatures (80–160 °C) for 1 h. Then, the samples were extracted with ethanol at room temperature. The corresponding results indicate that the total antioxidant capacity and metal chelating decreased, however the radical scavenging activity, the total flavonoid and polyphenol contents increased with the temperature.	Polyphenols and flavonoids (quercetin and ruthin).	[85]

## Abbreviations

aa_2_	Amino acid
BN	Boron nitride
BzSH	Benzylthiol
CQDs	Carbon quantum dots
DIPP-aa_1_	*N*-Phosphoamino acid
DHPs	Dihydropyridines
DHPDs	Dihydropyridinones
DHPMs	Dihydropyrimidinones
FIR	Far infrared irradiation
FRH	Far infrared–treated rice hull
FT-IR	Fourier transform infrared spectroscopy
GO	Graphene oxide
*i*-PropSH	Isopropanethiol
*n*-BuSH	*n*-Butanethiol
MCR	Multicomponent reaction
MH	Mantle heating
MIR	Middle infrared irradiation
MM	Mechanical milling
MW	Microwave irradiation
NIR	Near infrared irradiation
PhSH	Thiophenol
TAFF	Tonsil Actisil FF
US	Ultrasound
